# Time to go: neural crest cell epithelial-to-mesenchymal transition

**DOI:** 10.1242/dev.200712

**Published:** 2022-07-29

**Authors:** Tess A. Leathers, Crystal D. Rogers

**Affiliations:** Department of Anatomy, Physiology, and Cell Biology, UC Davis School of Veterinary Medicine, 1089 Veterinary Medicine Drive, Davis, CA 95616, USA

**Keywords:** Neural crest, EMT, Morphogens, Cadherins, Transcription factors, Post-translational modifications

## Abstract

Neural crest cells (NCCs) are a dynamic, multipotent, vertebrate-specific population of embryonic stem cells. These ectodermally-derived cells contribute to diverse tissue types in developing embryos including craniofacial bone and cartilage, the peripheral and enteric nervous systems and pigment cells, among a host of other cell types. Due to their contribution to a significant number of adult tissue types, the mechanisms that drive their formation, migration and differentiation are highly studied. NCCs have a unique ability to transition from tightly adherent epithelial cells to mesenchymal and migratory cells by altering their polarity, expression of cell-cell adhesion molecules and gaining invasive abilities. In this Review, we discuss classical and emerging factors driving NCC epithelial-to-mesenchymal transition and migration, highlighting the role of signaling and transcription factors, as well as novel modifying factors including chromatin remodelers, small RNAs and post-translational regulators, which control the availability and longevity of major NCC players.

## Abstract (precis)

**Summary:** Neural crest cells are an embryonic population of migratory stem cells that form many derivatives. This Review summarizes our current understanding of the molecular mechanisms driving their epithelial-to-mesenchymal transition.

## Introduction

**Fig. 1.**
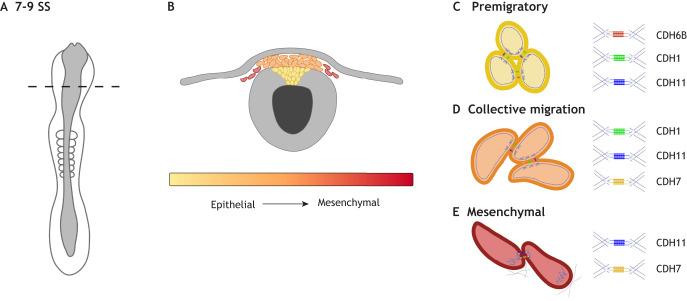
**Stages of NCC EMT in avians.** (A) NCC EMT occurs in three main stages in avian embryos: delamination, collective migration and mesenchymalization. A shows a schematic of a whole-mount chicken embryo at cranial NCC EMT stage [7-9 somite stage (SS)]. Dashed line indicates axial level in B. (B) Depiction of NCC population in premigratory epithelial to migratory mesenchymal cells during EMT and early migration. (C) Premigratory NCCs (yellow cells) express CDH6B (red) during neural tube closure and before delamination (Coles et al., 2007; Taneyhill et al., 2007), and CDH1 (green) and CDH11 (blue) as they undergo EMT (Rogers et al., 2018; Manohar et al., 2020). Premigratory NCCs delaminate from the epithelial neural tube, maintaining CDH1 and CDH11, and begin to downregulate CDH6B while maintaining adhesive contacts and nonpolarized actin (purple ring) localization (Manohar et al., 2020; Rogers et al., 2018). (D) NCCs then begin collective migration (orange cells) out of and away from the neural tube, maintaining CDH11 adhesive contacts and beginning to form transient CDH7 (yellow) adhesive contacts (Manohar et al., 2020). During this process, actin begins to localize to the leading edge of NCCs. (E) NCCs then fully mesenchymalize (red cells) and individually migrate through the extracellular matrix with transient CDH11 and CDH7 adhesive contacts and polarized actin localization to the leading edge (Rogers et al., 2018; Wu and Taneyhill, 2019). These steps differ slightly between organisms (Hamburger Hamilton stage 9 avian depicted). Figure created using BioRender.com.

Neural crest cells (NCCs) are transient embryonic stem cells that give rise to craniofacial bone and cartilage, portions of the sensory system, and cranial nerves, among other derivatives (Martik and Bronner, 2017; Mendez-Maldonado et al., 2020; Santagati and Rijli, 2003; Taneyhill et al., 2007). In most vertebrates, induction and specification of NCCs happens rapidly within, or adjacent to, the developing dorsal neural tube. NCC specification is marked by dynamic gene expression and changes in protein localization. These changes drive structural and patterning transitions allowing for tightly adherent NCCs to separate from their epithelial neighbors in an epithelial-to-mesenchymal transition (EMT) and migrate to distant sites in the developing embryo (Dady and Duband, 2017; LaBonne and Bronner-Fraser, 1998a; Mayor et al., 1995; Rogers and Nie, 2018; Selleck and Bronner-Fraser, 2000) (Fig. 1). Both historical and recent studies have defined a baseline gene regulatory network (GRN) of factors controlling the formation, migration and differentiation of NCCs, but the control of these processes is more complex, with epigenetic and environmental components. Here, we detail some of the recent discoveries defining new nodes and supporting traditional factors in the control of NCC EMT.

The well-established pathway of NCC development begins with morphogens that activate the expression of transcription factors, which in turn drive the expression of genes that modulate cell polarity and adhesion. These changes then drive NCC EMT. However, new research has revealed that emerging factors modulate this traditional pathway of NCC development. In addition to the established NCC GRN, epigenomic remodeling, post-transcriptional control, post-translational control and membrane remodeling impact NCC development. These newly discovered modulators are necessary for the proper expression of signals driving NCC EMT.

Along with transcriptional changes come physical changes in the organism. NCCs reduce their cell-cell and cell-basement membrane adhesion, lose or modify apicobasal polarity, and gain mobility through the remodeling of their cytoskeleton (Gouignard et al., 2018; Wu and Taneyhill, 2019). As NCC delamination begins, NCCs must detach from the neuroepithelium and, at least in chick, the basement membrane is remodeled to form a channel through which NCCs can migrate (Hutchins and Bronner, 2018). Migratory NCCs then respond to localized environmental cues as they move through the extracellular matrix (Kerosuo and Bronner-Fraser, 2012).

This Review includes many studies in chick due to their popularity as a model for NCC research, although we do mention other species where appropriate. These recent studies mainly focus on cranial NCC regulation, which can differ from signaling and morphological changes in the trunk region of embryos. Although some findings may be conserved across axial levels and species, care should be taken in considering the similarities and differences of these processes. Additional consideration of *ex vivo* and *in vitro* study of NCC development is considered in Box 1.

## Classical factors in NCC formation and EMT

### Signaling pathways

In vertebrates, NCC induction begins as early as gastrulation, driven by bone morphogenetic protein (BMP), fibroblast growth factor (FGF), Notch and Wnt signals from the ectoderm and mesoderm (Bonstein et al., 1998; Cheung et al., 2005; LaBonne and Bronner-Fraser, 1998b). Although BMP is most established as an NCC inducer (Garnett et al., 2012; Reichert et al., 2013; Shi et al., 2011), recent work in chick embryos identified that elevated BMP signaling is also necessary during NCC delamination and migration (Piacentino et al., 2021), as well as completion of migration (Rekler and Kalcheim, 2022). Similarly, Wnt modulators have come to the forefront as EMT regulators in recent years. In *Xenopus laevis* (frog), β-catenin, the main effector downstream of canonical Wnt signaling, is present in premigratory but not migratory NCCs, suggesting that Wnt signaling must be inhibited before NCCs can migrate from the neural tube (Maj et al., 2016). Wnt inhibition is mediated by scaffold proteins Dact1 and Dact2 (Rabadan et al., 2016) and the secreted molecule Draxin in developing chick NCCs (Hutchins and Bronner, 2018). FGF signaling is also downregulated to allow for chick NCC specification and EMT (Martínez-Morales et al., 2011). Notch signaling has not yet been demonstrated as necessary for NCC EMT, but it does establish the definitive roof plate from the dorsal neural tube from which NCCs emigrate – at least in amniotes (Ofek et al., 2021). With these studies, the once simple model of morphogens driving transcriptional regulator expression in NCCs has become more complex, suggesting waves of signaling rather than finite signals. More information on the roles of signaling pathways in NCC development can be found in detailed reviews (Artinger and Monsoro-Burq, 2021; Rogers et al., 2012; Rogers and Nie, 2018; Williams and Bohnsack, 2019).

### Transcriptional control of EMT and migration

During NCC induction, the aforementioned morphogens activate the expression of a host of transcription factors called neural plate border (NPB) specifier genes (Williams et al., 2022). The NPB forms between the neural tube and non-neural ectoderm and gives rise to both NCCs (on the medial side) and cranial placodes (on the distal side) (Plouhinec et al., 2017). The transcription factors that act as NPB-specifier genes vary depending on the species. *Zic1*, *Msx1a* and *Pax3a* form the NPB in fish (Garnett et al., 2012; Seo et al., 1998), *Zic1*, *Msx1* and *Pax3* in *X. laevis* (Maczkowiak et al., 2010; Monsoro-Burq et al., 2005), *Msx1* and *Pax7* mark the NPB in chick (Basch et al., 2006; Streit and Stern, 1999), and both *Pax3* and *Pax7* in concert with Zic genes are required for the formation of NCC derivatives in mice, although the two Pax homologs appear to have functional redundancy (Bellchambers et al., 2021; Mansouri et al., 1996). Throughout induction, the future NCCs remain adhered to the neuroepithelium and non-neural ectoderm in an epithelial state.

**Fig. 2.**
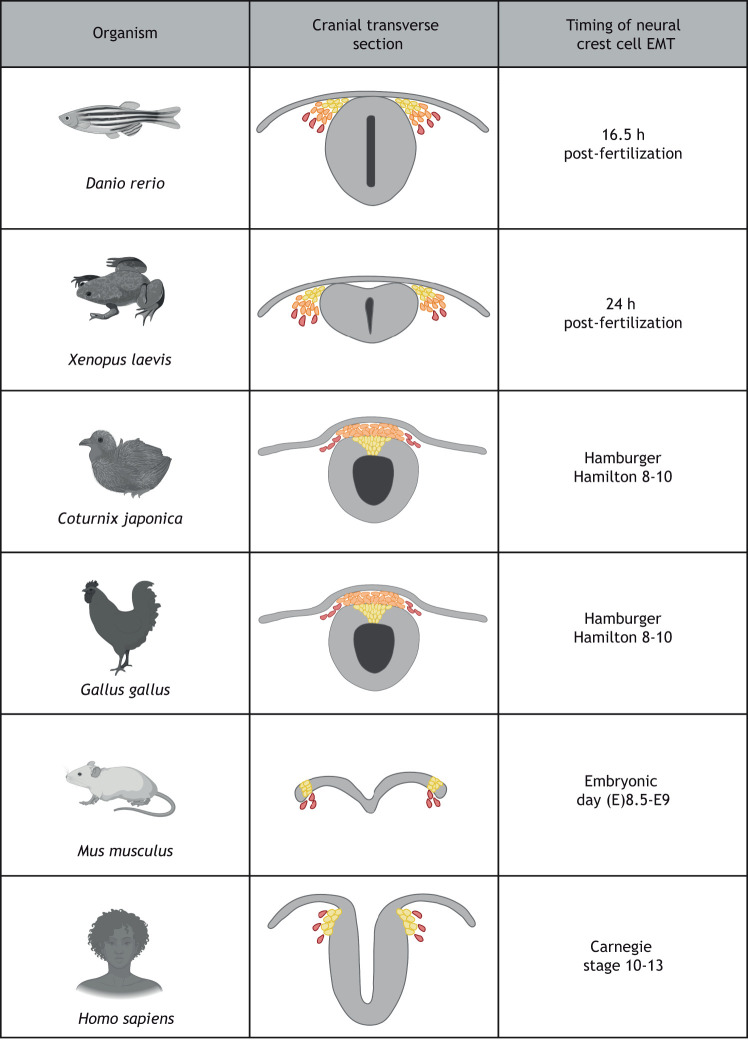
**Organismal differences in NCC EMT.** The process of NCC EMT varies based on the organism as well as at different axial levels. In avian species, NCCs must delaminate from the neural tube before emigrating (Monroy et al., 2022). In other species, such as zebrafish (Rajan et al., 2018; Wang et al., 2019) and frogs (Lee and Saint-Jeannet, 2011), NCCs arise adjacent to the neural tube before emigrating laterally. Mouse NCCs lack collective migration and instead quickly mesenchymalize for individual migration before the neural tube has closed (Lee et al., 2013). Human cells appear to migrate similarly to both rodents and avians (Betters et al., 2010). Figure created using BioRender.com.

As development proceeds, the NPB specifier proteins activate NCC-specifying transcription factors (NCC specifiers), including Snail, FoxD and SoxE family members, which are relatively conserved in spatiotemporal and hierarchical expression (Green et al., 2015; Simoes-Costa and Bronner, 2015) across vertebrates (Roellig et al., 2017; Seal and Monsoro-Burq, 2020; Stundl et al., 2021). However, their specific regulatory and coding sequences, as well as their functions, have not all been studied in multiple organisms (Monroy et al., 2022; Prescott et al., 2015). These transcription factors are responsible for initiating NCC EMT by directing changes in cell polarity and cell adhesion. They allow cells to delaminate from their neuroepithelial neighbors and collectively exit the neural tube in chick (Cheung and Briscoe, 2003; Cheung et al., 2005), quail (Sakai et al., 2006, 2005), human (Betters et al., 2010) and rodents (Betters et al., 2018; Lee et al., 2013), or collectively migrate ventrolaterally from the NPB in *X. laevis* (Maczkowiak et al., 2010; Plouhinec et al., 2017) and zebrafish (Fig. 2). In mouse and rabbit, NCCs begin to migrate before the neural tube has even closed (Betters et al., 2018; Lee et al., 2013) (Fig. 2).

To accomplish these changes in cell polarity and adhesion, NCC specifiers directly regulate the transcription of genes that code for calcium-dependent adhesion proteins called cadherins. These cadherin proteins drive changes in complex networks that cross over in feedback and feedforward loops. SNAI2 is a zinc-finger transcription factor that directly represses cadherin 6B (*CDH6B*; also known as *CDH6*) expression in chick, allowing NCCs to lose cell-cell adhesion and delaminate from the neural tube (Schiffmacher et al., 2014, 2016; Taneyhill et al., 2007). SNAI2 regulates E-cadherin (*CDH1*) gene expression in both human embryonic stem cells (Aban et al., 2021) and prostate cancer cells (Xie et al., 2014) to drive migration. Furthermore, work in *Xenopus* has demonstrated that SNAI2 may also interact with the Polycomb repressive complex to regulate *CDH1* expression to control NCC EMT (Aban et al., 2021; Tien et al., 2015; Xie et al., 2014).

The winged-helix transcription factor FoxD3 functions to maintain stemness in chick and is differentially regulated in distinct NCC subpopulations (Simoes-Costa et al., 2012). The expression of *FoxD3* is controlled by multiple factors including the Wnt signaling pathway in zebrafish and chick (Costa et al., 2021; Simoes-Costa et al., 2015), Pax3 and Zic1 in *X. laevis* (Plouhinec et al., 2014) and Cdx4 in the zebrafish trunk (Rocha et al., 2021). FoxD3 regulates tetraspanin 18 (*Tspan18*) expression in the chick cranial NCC (Fairchild and Gammill, 2013). As TSPAN18 post-translationally maintains CDH6B protein levels in the chick dorsal neural tube, its downregulation by FoxD3 promotes cranial NCC EMT (Fairchild and Gammill, 2013).

It is thought that the SoxE family of transcription factors (containing Sox8, Sox9 and Sox10) have allowed NCCs to diversify their tissue derivatives during chordate evolution (Schock and LaBonne, 2020). The timing of *Sox8* expression varies among species: in chicken (Buzzi et al., 2021 preprint) and zebrafish (Yan et al., 2005) it is expressed after *Sox9* and *Sox10* and is associated with ear development (Buzzi et al., 2021 preprint; Okamoto et al., 2018). However, in *Xenopus Sox8* is expressed before the other SoxE genes and loss of *Sox8* delays NCC specification (O'Donnell et al., 2006). In avians, *Sox9* is upregulated during NCC specification before EMT (Khudyakov and Bronner-Fraser, 2009; Monroy et al., 2022). In quail, SOX9 interacts with SNAI2 protein to activate *Snai2* expression in a feedforward loop (Sakai et al., 2006). Work in chick demonstrated that SNAI2 then represses *CDH6B* expression during EMT to promote NCC delamination (Cheung and Briscoe, 2003; Liu et al., 2013; Taneyhill et al., 2007). Unlike *Sox8* and *Sox9*, *Sox10* is expressed at the onset of chick NCC delamination and remains active in migrating NCCs (McKeown et al., 2005; Monroy et al., 2022). In addition to driving differentiation of melanocyte and oligodendrocyte fates, SOX10 also functions to maintain NCC stemness in rodents (Kelsh, 2006). The literature lacks evidence of whether SoxE proteins regulate changes in cell-cell adhesion directly to drive NCC EMT.

Transcription factors bind to enhancers to modulate basal transcriptional levels of their target genes (Gandhi and Bronner, 2021). Recent studies have found that several specific enhancers play important roles in modulating the expression of key NCC factors. Work in mice has demonstrated that mutations in an extreme long-range enhancer that controls stage-specific *Sox9* expression in cranial NCCs causes Pierre Robin Syndrome (Long et al., 2020). In chick, the protein complex YAP-TEAD binds to tissue-specific enhancers to drive the expression of EMT factors in NCCs (Bhattacharya et al., 2020). There is much opportunity for continued study of the role of individual and combinatorial enhancers in NCC development. A recent reconstruction of the chick cranial NCC GRN has uncovered new super-enhancers that regulate NCC at EMT stages (Williams et al., 2019). Moreover, the NCC GRN has been investigated in lamprey to uncover its ancestral state using assay for transposase-accessible chromatin with high-throughput sequencing (ATAC-seq) analysis, to reveal cis-regulatory elements involved in NCC specification, which may be conserved across species, such as enhancers for *Tfap2B*, *SoxE* and *Hoxa2* (Hockman et al., 2019)*.*

Together, these studies show that NCC induction begins with morphogen-induced expression of NPB specifier genes, including those of the Msx, Pax and Zic families, depending on the species. NPB specifier genes then activate NCC specifiers, including those from the Snail, FoxD and SoxE families, to regulate downstream effectors such as cadherins. Subsequent changes in cell polarity and adhesion allow for the initiation of NCC EMT, whereby NCCs become migratory and form NCC-derived tissues.

## Cadherin-based cell adhesion changes during NCC EMT

**Fig. 3.**
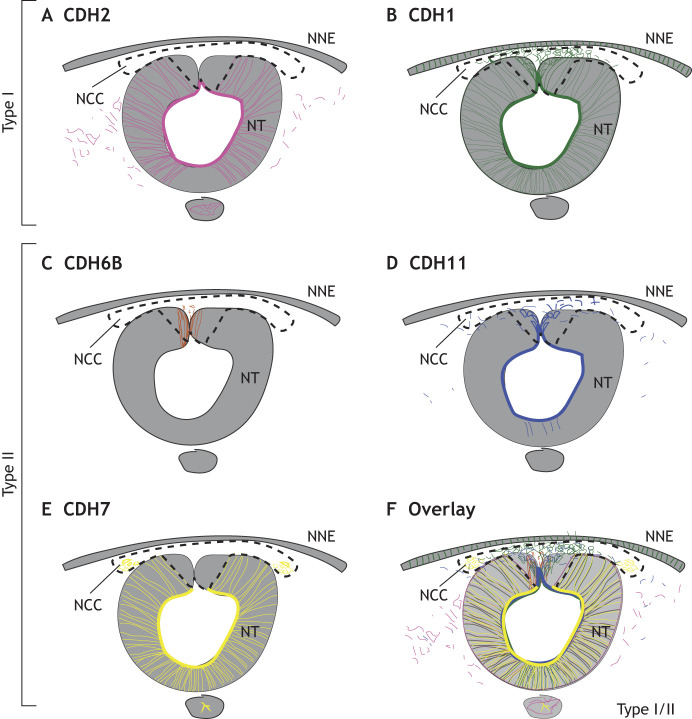
**Cadherin localization and specificity in chick embryos.** Work in chicken embryos has demonstrated dynamic changes in type I and II cadherin localization during neural crest cell (NCC) epithelial-to-mesenchymal transition (EMT). (A) CDH2 is expressed in the neural tube (NT) but is removed from NCCs during neural tube closure (Dady et al., 2012; Rogers et al., 2018). (B) CDH1 is expressed throughout the neural tube and is strongly expressed in premigratory NCCs (Dady et al., 2012; Rogers et al., 2018). (C) CDH6B is expressed in the dorsal neural folds during NCC induction and specification, and is downregulated prior to EMT (Coles et al., 2007; Strobl-Mazzulla and Bronner, 2012). (D) CDH11 is expressed in the neural tube but is upregulated in collectively migrating NCCs (Manohar et al., 2020). (E) CDH7 is upregulated as NCCs leave the neural tube and migrate ventrolaterally (Wu and Taneyhill, 2019). (F) Overlaying the diagrams demonstrates clear overlapping and distinct domains of expression for each cadherin molecule during NCC EMT. Dashed outline indicates dorsal neural tube region where premigratory NCCs originate and areas outside of the neural tube to which collective NCCs migrate after delamination. NNE, non-neural ectoderm.

A major role of transcription factors during NCC EMT is to regulate the dynamic expression of genes encoding cadherin proteins. Cadherins are calcium-dependent transmembrane proteins that interact with α-, β- and δ-catenin proteins intracellularly and regulate cell-cell adhesion during development (Stepniak et al., 2009). Classical cadherins can be divided into two types in developing NCCs (Fig. 3). Type I cadherins, such as epithelial (CDH1) and neural cadherin (CDH2), are expressed in the developing neural tube in chick embryos but, in EMT-stage cranial NCCs, CDH2 is mostly absent and CDH1 is upregulated (Dady et al., 2012; Rogers et al., 2018). In contrast to chick, frog (Bahm et al., 2017; Kotini et al., 2018; Scarpa et al., 2015) and zebrafish (Piloto and Schilling, 2010; Powell et al., 2015) NCCs appear to require CDH2 for normal migration, whereas the evidence for CDH1 in this process differs between studies (Huang et al., 2016).

Type II cadherins consist of proteins expressed in both premigratory and migratory NCCs. CDH6B is expressed during chick neural tube closure and is downregulated by SNAI2 to allow for NCC delamination (Coles et al., 2007; Padmanabhan and Taneyhill, 2015; Park and Gumbiner, 2010; Schiffmacher et al., 2014, 2016). Cadherin 11 (CDH11) is upregulated in premigratory NCCs and is necessary for NCC migration and survival in *X. laevis* (Kashef et al., 2009; Langhe et al., 2016; Mathavan et al., 2017; McCusker et al., 2009) and chick (Manohar et al., 2020) embryos. Cadherin 7 (CDH7) is the least well-studied type II cadherin protein, but it is upregulated in migratory NCCs and functions to pattern both the neural tube and differentiating NCCs in chick (Chalpe et al., 2010; Prasad and Paulson, 2011; Wu and Taneyhill, 2019).

Multiple transcription factors regulate the expression of genes coding for type I and II cadherin proteins. Cadherin proteins are localized to adherens junctions in epithelial cells, interacting with the actin cytoskeleton, but are capable of intracellular signaling in chick (Schiffmacher et al., 2014; Taneyhill and Schiffmacher, 2017). Cadherins also regulate EMT in cancer (Campbell and Casanova, 2016). However, there is very little known about how cadherin gene and protein expression is controlled during NCC specification and EMT. Chick *CDH2* enhancers contain SOX2 binding sites, suggesting a potential regulatory loop between SOX2 and *CDH2* expression (Matsumata et al., 2005). Their complementary expression, requirement for neural cell types and absence from the dorsal neural tube all support a mutual regulatory relationship. An inhibitory regulatory loop has been established between the EMT-driving transcription factor SNAI2 and *CDH6B*, linking cell adhesion to intracellular signaling (Schiffmacher et al., 2016; Taneyhill et al., 2007). In addition, SIP1 (ZEB2) modulates the CDH2/CDH1 reciprocal axis during NCC EMT (Rogers et al., 2013, 2018; van Grunsven et al., 2003).

Recently, it has been shown that cadherin proteins are post-translationally regulated. In chick cranial NCCs, metalloproteinases (such as ADAM10 and ADAM19) disassemble cadherin-based junctions, creating CDH6B N-terminal fragments (Schiffmacher et al., 2014). These fragments enhance proteolytic activity, reducing structural proteins laminin and fibronectin in the basement membrane and promoting delamination of NCCs. Similarly, the matrix metalloproteinase MMP14 is necessary for *X. laevis* NCC EMT, possibly through its reduction of cadherin levels (Garmon et al., 2018). CDH11 and its cleavage product EC1-3 are also implicated as regulators of NCC migration in *X. laevis* (Abbruzzese et al., 2016; Li et al., 2018; Mathavan et al., 2017; McCusker et al., 2009), and chick (Manohar et al., 2020).

Through the process of EMT, NCCs go from a stationary, adherent epithelial state to a migratory, transiently adherent and subsequently invasive mesenchymal state. To mediate these changes, most vertebrates undergo a switch in expression from type I cadherins before delamination to type II cadherins at the onset and during EMT for proper cellular interactions. These adhesion switches are controlled, in part, by transcription factors that are conserved across vertebrate species. Further research into the transcriptional and post-translational regulators of cadherins and their cleavage products may demonstrate an even greater refinement of structural remodeling at the onset of NCC migration. Understanding the multitude of mechanisms controlling NCC EMT and migration provides relevant information for a better understanding of both developmental processes and disease states.

## Emerging regulators of NCC EMT

### Epigenetic modifications and chromatin remodeling

Dynamic changes in chromatin accessibility are controlled by the action of chromatin modifiers including methyltransferases and demethylases. The methylation state of a histone protein can define whether a transcription site is active. By governing methylation states of developing NCCs, methylation modulators initiate or prevent the transcription of key factors at important developmental time points. A short summary of discoveries are highlighted below, but in-depth information about epigenetic modifications and chromatin remodeling in NCCs from multiple axial levels and in different organisms has been previously reviewed (Berube-Simard and Pilon, 2019; Hovland et al., 2020; Prasad et al., 2012; Schock and LaBonne, 2020; Yan et al., 2021).

#### Demethylases

Jumonji domain (Jmj)-containing proteins are a group of demethylases that regulate transcriptional activity mainly through the demethylation of lysines (Meng et al., 2018). These proteins play an important role in NCC development. During NCC specification in chick embryos, *KDM4A* (*JMJD2A*) is expressed in the forming neural folds, and blocking KDM4A translation dramatically reduces the expression of definitive NCC genes *Sox10*, *Snai2* and *Sox9* (Strobl-Mazzulla et al., 2010). Similarly, loss of the lysine-specific histone demethylase 5C (KDM5C) downregulates expression of NCC specifiers *Twist*, *Snai2*, *Sox8* and *Sox10* in *X. laevis* (Kim et al., 2018). Interestingly, the loss of KDM5C has no effect on *Sox9* expression, which may explain the absence of cranial cartilage deformities in KDM5C-deficient frogs. Also in *X. laevis*, *KDM3A* is expressed from early embryonic stages to tadpole stage, with a dramatic increase in expression during neurula stage (Lee et al., 2019). Knockdown of *KDM3A* impairs NCC migration, which could be explained by changes in the expression of factors that regulate mesoderm formation, cell adhesion and metabolic processes (Lee et al., 2019).

#### DNA methyltransferases

DNA methyltransferases add methyl groups to DNA using S-adenosyl methionine as the methyl donor. DNA methyltransferase 3B (DNMT3B) is a *de novo* methyltransferase that establishes DNA methylation patterns during embryonic development (Gagliardi et al., 2018). These methylation patterns are maintained throughout multiple rounds of cell division by a Dnmt1-mediated copying mechanism in order to create heritable epigenetic marks on the genome (Li and Zhang, 2014). DNMT3B modulates the activity of NCC factors at key time points. Knockdown of DNMT3B in chick embryos extends NCC production and emigration from the neural tube due a defect in *Sox10* promoter methylation (Hu et al., 2014). *Sox10* is expressed in NCCs after they are specified, and its regulatory region is methylated after NCC emigration in normal embryos, but after DNMT3B knockdown, the methylation mark is reduced (Hu et al., 2014). DNMT3B knockdown in chick also significantly upregulates the expression of NCC specifier genes (e.g. *TfAp2A*, *Sox9*, *Sox10*, *Snai2* and *FoxD3*) and downregulates *CDH2* (Hu et al., 2014). In addition to its role in regulating *Sox10* expression, DNMT3B functions in a negative-feedback loop between miR-203 and *Snai2* in chick (Sanchez-Vasquez et al., 2019). miR-203 plays an important role as a regulator of NCC delamination timing because ectopic miR-203 inhibits NCC migration, whereas loss of miR-203 promotes premature NCC delamination (Sanchez-Vasquez et al., 2019). Another DNA methyltransferase, DNMT3A, is expressed in the NPB and drives NCC specification by repressing neural tube markers *Sox2* and *Sox3* (Hu et al., 2012). *DNMT3A* expression is mediated by a *Pou3f1-*miR-29b*-DNMT3A* axis that determines NCC versus neural tube fates (Xi et al., 2017). These studies suggest that both DNA methyltransferases and miRNAs play important roles in NCC specification and that studies must extend to other modulators of NCC transcripts to further refine our understanding of the GRN.

Chromatin remodeling factors alter gene expression by either using covalent histone modifications, such as acetylation, or ATP-dependent chromatin remodeling. Altering chromatin changes the accessibility of DNA molecules to transcription factors, and some chromatin remodelers also regulate the expression of NCC genes. One such chromatin remodeler, Hmga1, has two important roles in chick. First, Hmga1 activates the expression of the NCC progenitor marker *Pax7* at the NPB. Second, Hmga1 downregulates Wnt signaling, thereby modulating the interaction of Wnt with its downstream targets, such as *Snai2*. Loss of Hmga1 reduces cranial NCC migration from the neural tube, but this phenotype is rescued by the addition of β-catenin (Gandhi et al., 2020b). FoxD3, a pioneer transcription factor that can recruit chromatin remodeling factors, primes genes for NCC specification and acts as a repressor to control NCC migration and differentiation in zebrafish. Specifically, it represses multiple genes involved in NCC migration and differentiation, including *nrp2a*, *nrp1b* and *slit1a* (Lukoseviciute et al., 2018). Similarly, TFAP2 is associated with permissive chromatin states in chick NCCs (Rothstein and Simoes-Costa, 2020). TFAP2 functions in a heterodimer with TFAP2C during gastrulation, activating NCC inducers. TFAP2A then switches partners to heterodimerize with TFAP2B as neurulation begins. *TFAP2B* overexpression significantly decreases *TFAP2C* expression, and premature expression of *TFAP2B* significantly increases definitive NCC marker expression, indicating that TFAP2B likely represses TFAP2C to drive NCCs to specification (Rothstein and Simoes-Costa, 2020). These studies show that chromatin remodeling factors often play multiple regulatory roles throughout NCC development, and that we must consider the chromatin environments in addition to other highly studied regulators to fully understand NCC EMT.

### Nutrients and environment

The mechanisms linking environmental factors to NCC development are highly understudied. Here, we discuss what is currently known and where the field can be expanded.

#### Folic acid

Folic acid/folate is a B vitamin, deficiency of which during development leads to anomalies in neural tube closure, heart formation and craniofacial development (Alata Jimenez et al., 2018; Harris and Juriloff, 2010; Karunamuni et al., 2014). Folate is the main source of methyl groups for DNA and histones, and thus knockdown of its transporters in chick embryos unsurprisingly reduces the abundance of histone H3 lysine and DNA methylation (Alata Jimenez et al., 2018). Knockdown of folate transporters also leads to ectopic expression of the stemness and neural progenitor marker *Sox2* at the expense of definitive NCC markers, caused by failure of DNA methylation (Alata Jimenez et al., 2018). In both *Xenopus* and chick embryos, the border between *Sox2*- and *Sox3*-expressing neural progenitor cells and NCCs is tightly regulated and, therefore, direct or indirect alterations in *Sox2* expression via folate deficiency cause NCC defects (Hu et al., 2012; Rogers et al., 2009).

#### Retinoic acid

The vitamin A metabolite retinoic acid (RA) acts as a morphogen in early development controlling embryonic patterning and NCC migration (Thompson et al., 2019). Recent work in chick embryos has demonstrated that RA works in concert with other morphogens such as BMP and FGF to modulate NCC migration. Namely, inhibition of RA in the neural tube prevents upregulation of BMP inhibitors, thereby prolonging BMP signaling and NCC emigration from the neural tube (Rekler and Kalcheim, 2022). Moreover, RA acts in an opposing gradient with FGF signaling along the anteroposterior axis of chick embryos to control the timing of NCC EMT (Martinez-Morales et al., 2011). Work in zebrafish has established that RA signaling regulates both migration and differentiation of NCCs at both cranial and vagal axial levels into craniofacial structures and enteric nerves (Chawla et al., 2018; Reijntjes et al., 2007; Uribe et al., 2018).

More work is needed to understand the extent to which environmental factors can affect NCC formation and development. For example, induced inflammation reduces NCC EMT and causes craniofacial defects in chick (Li et al., 2022). However, embryonic exposure to non-steroidal anti-inflammatories in zebrafish, mouse and chick causes abnormal cranial and vagal NCC formation and migration, leading to craniofacial and enteric defects (Parmar et al., 2021; Schill et al., 2016). These works suggest that there are additional understudied and less common pathways that may intersect with the more common NCC GRN pathways, such as Wnt, BMP or FGF pathways. Future work on understanding intersections between the major signaling pathways and those of lipid modifiers, intracellular enzymatic proteins, extracellular matrix molecules and others would greatly improve clarity in the field.

## Post-transcriptional control

Although most of the regulation governing NCC EMT has been studied at the transcriptional level, recent studies have shown that post-transcriptional modulators play an important role in the process of EMT. Post-transcriptional control involves modifications to mRNA before it is translated into protein. Further modulation of RNA stability, degradation and rate of translation is performed by a variety of small noncoding RNAs, including Piwi-interacting RNAs (piRNAs) and microRNAs (miRNAs), as well as RNA-binding proteins.

### Small noncoding RNAs

The Piwi family of Argonaute proteins is responsible for maintenance of stemness and protection against transposable elements, which endanger the genome by replicating and inserting themselves at new positions (Cenik and Zamore, 2011; Galton et al., 2021 preprint). piRNAs recognize transposable elements and target them for destruction by Piwi proteins. *Piwil1* is expressed at low levels throughout chick NCC development, with a peak in expression just before NCC EMT, and a reduction of Piwil1 in the dorsal neural tube prevents NCC emigration (Galton et al., 2021 preprint). Piwil1 downregulates expression of *Gallus gallus* early response to neural induction (*ERNI*), a transposable element-derived gene, but the mechanism by which these factors regulate NCC emigration is still unknown (Galton et al., 2021 preprint). However, an inverse relationship exists between *ERNI* and *Sox2* expression, in which *Sox2* is expanded in the absence of ERNI, and excess Sox2 inhibits NCC specification (Galton et al., 2021 preprint; Papanayotou et al., 2008; Streit et al., 2000). This established relationship may provide some explanation for why loss of Piwil1 prevents NCC emigration, but further investigation is still required.

miRNAs are well established as post-transcriptional gene regulators (Filipowicz et al., 2008). Knockdown of DICER, a key enzyme in miR-200a, miR-20a and miR-217 biogenesis, leads to a significant decrease in NCC marker expression and NCC derivative formation (Copeland et al., 2021; Huang et al., 2010; Nie et al., 2011; Song and Rossi, 2017; Zehir et al., 2010). Furthermore, only the addition of wild-type DICER can rescue the expression of NCC markers after DICER knockdown. These miRNAs target and post-transcriptionally repress components of the FGF pathway, which is necessary for NCC induction in several species (Copeland et al., 2021). Ectodermal explants from *X. laevis* embryos have revealed 11 miRNAs that are enriched in induced NCC tissue (Ward et al., 2018). Further functional studies investigating the role of these enriched miRNAs may reveal novel regulators of NCC specification and EMT. The field is ripe for continued studies of miRNA regulation during NCC EMT.

### RNA-binding proteins

Lin28a is an RNA-binding protein that promotes pluripotency and inhibits maturation of the let-7 family of miRNAs (Newman et al., 2008). Recent analysis of chick cranial NCCs has identified that *Lin28a* expression correlates with NCC stemness markers, which decrease during late stages of migration (Bhattacharya et al., 2018). Further gain- and loss-of-function experiments have found that constitutive expression of Lin28a leads to abnormal maintenance of stem cell factors and a subsequent delay in differentiation, whereas premature downregulation of Lin28a leads to increased let-7 miRNA and decreased expression of NCC stem markers (Bhattacharya et al., 2018). In fact, a Wnt-mediated stem cell niche may control the Lin28a/let-7 regulatory circuit because chick NCCs lose Wnt activation of Lin28a and gain let-7 miRNA expression as they migrate away from the neural tube (Bhattacharya et al., 2018). In *X. laevis* and zebrafish, ectopic expression of *Lin28a* in late stages prevents sympathoadrenal cell differentiation and accelerates NCC migration (Corallo et al., 2020). These findings point to the Lin28a/let-7 axis as a spatiotemporal regulator of NCC stemness, controlling onset of differentiation and determination of cell fate during NCC migration.

HuR (Elavl1) regulates proliferation and differentiation through its regulation of mRNA stability (Srikantan and Gorospe, 2012). HuR is enriched in avian embryos during NCC specification and loss of HuR results in a significant reduction in the expression of the NCC specifier *FoxD3*, its activator *Axud1* (*CSRNP1*) and the EMT regulator *Draxin*, causing premature NCC delamination from the neural tube (Chacon et al., 2021). Overexpression of exogenous *Draxin* rescues cranial NCC specification defects, implying that HuR maintains cranial NCC specification through its stabilization of *Draxin* (Chacon et al., 2021).

## Post-translational control

Post-translational modifications to proteins, such as phosphorylation, ubiquitylation and SUMOylation, modulate protein expression, localization and stability during development.

### Phosphorylation

Protein phosphorylation is a mechanism commonly employed by cells to modulate protein activity in processes such as cell signaling, gene expression and differentiation. Kinases attach phosphate groups to proteins, whereas phosphatases remove them. One such phosphorylation enzyme, AKT kinase, has been a recent subject of interest for its role in NCC migration. Work in *X. laevis* embryos has shown that the CDH11 extracellular domain cleavage product (EC1-3) stimulates phosphorylation of AKT, and that AKT is necessary for proper cranial NCC migration (Mathavan et al., 2017). Work in *Xenopus tropicalis* showed that the RNA helicase DDX3 regulates AKT kinase activity during neural induction (Perfetto et al., 2021). Loss of DDX3 decreases AKT activity and AKT-dependent inhibition of glycogen synthase kinase 3β (GSK3β), thereby reducing levels of GSK3β targets β-catenin and *Snai1*, which are necessary for NCC induction in *X. tropicalis* (Perfetto et al., 2021). GSK3β is also necessary for NCC migration in both *X. laevis* and mouse models, and loss of GSK3β leads to dysregulation of Rac1 and lamellipodia formation necessary for cell migration (Gonzalez Malagon et al., 2018). Endothelin signaling is necessary to phosphorylate the Cdc42 target ACK in mice, which is necessary for NCC migration into the cardiac outflow tract (Fritz et al., 2019). The Eph-Ephrin signaling pathway has also been implicated in the migration of cranial NCCs in *Xenopus*: binding of ephrinB2 to its receptor leads to its phosphorylation and disruption of its complex with Dsh and TBC1d24, increasing CDH1 expression on NCC membranes and disrupting NCC migration (Yoon et al., 2018).

### Ubiquitylation

Ubiquitylation is the process by which proteins are tagged with ubiquitin, marking them for degradation by the proteasome. Ubiquitin ligases mediate this process by recruiting an E2 ubiquitin-conjugating enzyme to transfer ubiquitin to a lysine on the target protein. Mice lacking the ubiquitin ligase Nedd4 have NCC defects, likely through Nedd4-mediated positive regulation of the NCC factors *Sox9*, *Sox10* and *FoxD3* (Wiszniak et al., 2013). In zebrafish, Nrarp blocks the ubiquitylation of Wnt pathway component LEF1 and its loss leads to defects in NCC migration and differentiation (Ishitani et al., 2005). Ubiquitylation-mediated control of NCC development has been vastly understudied, but future investigation into ubiquitylation as a mechanism regulating protein turnover during NCC migration may prove promising to understand the rapid changes that occur as the cells undergo EMT.

### SUMOylation

SUMOylation is a process by which SUMO, a small ubiquitin-like modifier protein, attaches to proteins to alter their functions. Early work in *X. laevis* embryos demonstrated that SUMOylation modulates the function of SoxE proteins, turning them into transcriptional repressors (Lee et al., 2012). Recent work has shown that the activity of the zinc-finger family transcription factor, ZIC5, is modulated after SUMOylation, and lack of SUMOylation causes NCC defects in mice (Ali et al., 2021). ZIC5 activates the expression of the NCC specifier *FoxD3* and interacts as a co-factor with TCF/LEF proteins to repress Wnt signaling, but SUMOylation of ZIC5 reduces the ZIC5/TCF/LEF complex and instead favors increased *FoxD3* expression (Ali et al., 2021). SUMOlyation mediates the function of additional NCC transcription factors. In chick, SOX9 must be phosphorylated and SUMOylated to interact with SNAI2 and promote NCC delamination (Liu et al., 2013) and PAX7 must be SUMOylated for proper NCC specification (Luan et al., 2013).

## Basement membrane remodeling

The basement membrane is a specialized extracellular matrix that lines the outer, basal side of the neural tube. The basement membrane must be remodeled to form a channel between the neural tube and overlying epidermis before NCCs can initiate the EMT process of delamination in some organisms. Studies have only recently begun to uncover the molecular dynamics of basement membrane remodeling during NCC EMT.

One major component of the basement membrane is the structural protein, laminin. EMT involves three stages of basement membrane protein laminin remodeling: regression, expansion and channel formation. Work in chick embryos has shown that the Wnt antagonist Draxin played multiple roles in regulating laminin remodeling in cranial NCCs (Hutchins and Bronner, 2018). Perturbation of Draxin expression at multiple steps blocks laminin remodeling, and this process is controlled by cytoplasmic RNA granules called ‘processing bodies’ to maintain a proper flux of Wnt signaling (Hutchins et al., 2021).

Matrix proteins are broken down by metal-assisted enzymes called matrix metalloproteinases (Nagase et al., 2006). MMP9 has been implicated as a regulator of cranial and trunk chick NCC EMT because MMP9 inhibition or overexpression reduces or enhances NCC migration, respectively (Monsonego-Ornan et al., 2012). MMP9 likely modulates NCC EMT through its degradation of the adhesion protein CDH2 and membrane component laminin (Monsonego-Ornan et al., 2012). Similarly, MMP2 activity is modulated by the cleaved N-terminal fragment of CDH6B and this interaction is necessary for NCC EMT (Schiffmacher et al., 2018). Work in *X. laevis* has demonstrated that the metalloproteinase ADAM13 regulates cranial NCC migration via cleavage of CDH11 (Abbruzzese et al., 2016) and modulation of Wnt signaling (Li et al., 2018). ADAM19 functions nonproteolytically in NCC specification by inhibiting the proteasomal degradation of ADAM13, adding another layer of complexity through protease–protease interaction (Li et al., 2018). As cadherin proteins are a major target of the NCC GRN factors and many are also post-translationally cleaved via MMPs, future work is necessary to characterize the differing roles of full-length versus cleaved fragments of these proteins in NCC EMT.

## Lipid modifications

Cell membranes are composed of lipids, including phospholipids, glycolipids and cholesterol. A recent screen of lipid-modifying genes during chick NCC EMT and migration identified that the sphingolipid-metabolizing enzyme nSMase2 (*Smpd3*) is differentially expressed over the course of NCC EMT (Piacentino et al., 2020 preprint). Knockdown of nSMase2 decreased Wnt and BMP signaling and subsequently downregulated downstream promigratory NCC factors. nSMase2 mediates plasma membrane activities, such as endocytosis of Wnt and BMP ligands, to activate pro-EMT factors, such as *Snai2* and *Sox9*, in chick (Piacentino et al., 2020 preprint). In addition to endocytosis of extracellular morphogens, transmembrane proteins are also endocytosed. For example, CDH6B is removed from premigratory chick NCCs through clathrin-mediated endocytosis and macropinocytosis (Padmanabhan and Taneyhill, 2015). Pharmacological inhibition of these processes in chick NCC explants inhibits NCC migration (Padmanabhan and Taneyhill, 2015). Finally, during NCC migration, cells form lamellipodia at the leading edge (Li et al., 2020). Recent live imaging of chick NCCs has shown that NCC membranes are remodeled through macropinocytosis and shuttling of F-actin to the lamellipodium (Li et al., 2020).

## Conclusion

The current framework outlining the molecular mechanisms driving NCC EMT and migration focuses strongly on a central GRN controlled by dynamic changes in transcription factors that regulate the expression of downstream adhesion factors. However, recent work has shown that NCC migration is regulated at multiple levels by diverse factors, which illustrates the true complexity of this process (summarized in Table 1). Modulators from the epigenomic to post-translational levels play key roles in regulating NCC EMT.

**Table 1.**
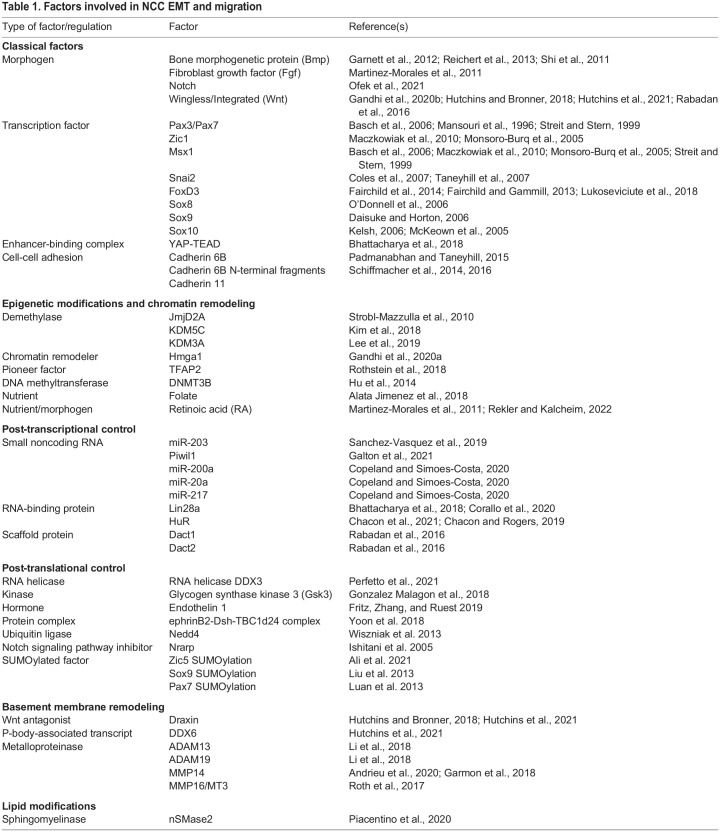
Factors involved in NCC EMT and migration

Continued studies in animal models are essential to drive the field forward and to identify new connections between the factors that drive dynamic transitions in cell states. Although we have discussed studies in several model organisms in parallel, it is worth noting that studies of NCC EMT cannot always be applied broadly. Embryo gastrulation is vastly different between popular models, such as chick, frog, zebrafish and mice (Stower and Bertocchini, 2017). It follows that NCC development during neurulation and migration stages may exhibit key morphological and molecular differences between organisms. Even between similar species, timing and localization of conserved NCC transcription factors differs (Monroy et al., 2022). Traditionally, it is thought that transcription factors are conserved across vertebrate species, but even with major drivers such as Pax3 versus Pax7, differences exist. Although frog and fish rely on Pax3 as a driver at the NPB (Maczkowiak et al., 2010; Seo et al., 1998), other aquatic animals like axolotls do not contain Pax3 in their genomes (Nowoshilow et al., 2018). The loss of a major NCC regulator in a vertebrate organism suggests that there may be parallel pathways controlling development. In addition, downstream effectors like cadherins appear to be functionally different or flipped between species. For example, the type I cadherin CDH2, which is dynamically modulated during NCC EMT, is downregulated at the onset of cranial NCC EMT in chick (Dady and Duband, 2017; Rogers et al., 2018), but it is necessary for NCC migration in frogs (Bahm et al., 2017). Moreover, NCC development, EMT and differentiation proceeds through different mechanisms at different axial levels, even within a given organism (Lallier et al., 1992; Simoes-Costa and Bronner, 2016). Future studies into the mechanisms regulating NCC EMT must take axial position and organismal differences into account, and side-by-side comparisons using the same tools will improve the clarity of conserved and divergent properties of NCCs. Additional consideration and information will be gained by analyzing information provided using both animal models and *in vitro* or *ex vivo* models (Box 1).

**Box 1. NCC development outside of the embryo**

In the current Review, we focus on detailing molecules and processes that have been identified as regulators of NCC *in vivo* using animal models. However, *ex vivo* and *in vitro* techniques for the study of NCC development have been pioneered in multiple laboratories. A popular method used to study NCC EMT and migration outside of the embryonic microenvironment (*ex vivo*) is the creation of NCC explants, where precursors to NCCs (dorsal neural tube or neural plate border) are dissected out of the embryo before EMT and are cultured on slides coated with extracellular matrix glycoproteins. Explants have been used to study murine (Baroffio et al., 1991), avian (Pfaltzgraff et al., 2012; Rogers et al., 2013) and amphibian (Cousin and Alfandari, 2018) NCC development. This technique allows closer inspection of cellular anatomy and processes in a 2D environment. Although multipotent NCCs intrinsically migrate and differentiate in explant conditions, another *ex vivo* culture method creates crestospheres, which maintain NCC multipotency long-term before differentiation, thus allowing researchers to investigate questions about pluripotency and pathology (Kerosuo et al., 2015; Mohlin et al., 2019). In addition to using *ex vivo* methods, true *in vitro* methods, such as NCCs derived from organoids (Abdel Fattah et al., 2021; Karzbrun et al., 2021; Lee et al., 2022) and induced pluripotent stem cell-derived NCCs, have been used to discover new information about pluripotency (Hackland et al., 2019; Prasad et al., 2020; Zalc et al., 2021), rare NCC-derived disorders (Bajpai et al., 2017; Okuno et al., 2017; Pauli et al., 2017) and novel information about epigenetic and transcriptional control of NCC genes (Long et al., 2020; Prescott et al., 2015). Further information on *in vitro* neural crest techniques is reviewed by Dupin et al. (2018).
